# The paradox of hypothyroidism: elevated cardiac biomarkers without coronary artery disease

**DOI:** 10.1530/EDM-24-0147

**Published:** 2025-10-28

**Authors:** Pooja Alipuria, Atush Alipuria

**Affiliations:** ^1^ Department of Internal Medicine, Yashoda Super Speciality Hospital, Kaushambi, Ghaziabad, India; ^2^ Department of Pulmonary Medicine, Yashoda Super Speciality Hospital, Kaushambi, Ghaziabad, India

**Keywords:** hypothyroidism, elevated CK-MB, thyroid, coronary artery disease

## Abstract

**Summary:**

This case report describes a 55-year-old male with hypothyroidism who presented with chest pain, elevated cardiac biomarkers (creatine kinase-MB (CK-MB) and troponin T), and abnormal electrocardiogram (ECG), findings suggestive of acute coronary syndrome (ACS). Despite clinical suspicion of myocardial ischemia, coronary angiography revealed no significant coronary artery disease. Profound hypothyroidism, confirmed by markedly elevated thyroid-stimulating hormone (TSH) and anti-thyroid peroxidase antibodies, was identified as the underlying cause of the cardiac biomarker abnormalities. Treatment with thyroxine resulted in clinical improvement and normalization of cardiac markers. This case underscores the importance of considering hypothyroidism in the differential diagnosis of elevated cardiac enzymes and ischemic symptoms in the absence of coronary artery disease.

**Learning points:**

- Hypothyroidism should be considered in patients presenting with ischemic symptoms and elevated cardiac biomarkers, in the absence of coronary artery disease.
- Elevated CK-MB and troponin in hypothyroidism may result from non-cardiac mechanisms, posing significant diagnostic challenges.
- Thyroid function tests should be routinely included in the diagnostic evaluation of patients with atypical presentations of myocardial injury.
- Timely diagnosis and management of hypothyroidism can help avoid misdiagnosis and unnecessary invasive interventions.

## Background

This case highlights the intersection of endocrinology and cardiology, showcasing the atypical presentation of hypothyroidism that mimics acute coronary syndrome. The unique aspect of this case lies in the elevated CK-MB and troponin levels in the absence of myocardial damage, which presented a significant diagnostic challenge. While hypothyroidism is common, its presentation with ischemic symptoms and ECG abnormalities is relatively rare. This case underscores the importance of considering thyroid dysfunction in the differential diagnosis of patients with atypical ACS presentations, emphasizing the need for thyroid function evaluation in such clinical scenarios. It provides valuable insights into the diagnostic complexity and clinical implications of hypothyroidism in patients with elevated cardiac biomarkers.

## Case presentation

A 55-year-old male presented to the emergency department with a 1-hour history of chest pain associated with breathlessness and sweating. He reported a similar episode the previous day, lasting approximately 40 min and relieved by nitrates. The chest pain was exertional, non-positional, and not related to trauma or fever.

His medical history was notable for dyspnea on exertion (New York Heart Association Class II) and generalized weakness for the past 5 days. He was an ex-smoker (smoking index 600; quit 8 months earlier) and consumed alcohol occasionally. There was no known family or personal history of cardiovascular disease.

**Figure 1:**
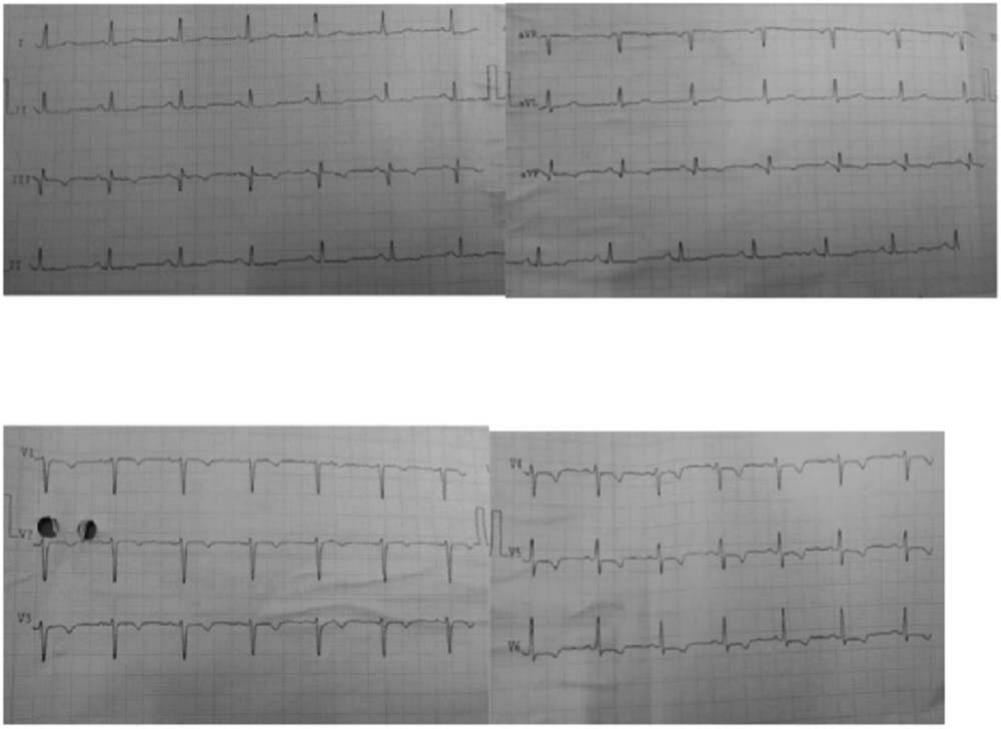
Electrocardiogram (ECG) showing sinus rhythm with symmetrical T-wave inversion in leads V1-V6, II, III, and aVF.

On examination, he was hemodynamically stable with a heart rate of 76 bpm and blood pressure of 150/90 mmHg. Cardiac auscultation was normal without murmurs or gallops. Respiratory and abdominal examinations were unremarkable. The initial ECG revealed sinus rhythm with symmetrical T-wave inversions in leads V1–V6, II, III, and aVF, suggestive of myocardial ischemia (Fig. 1).

## Investigation

Initial laboratory investigations showed a significantly elevated CK-MB (497 U/L) and positive troponin T levels (kit based), indicating myocardial injury. Total creatine kinase (CK) was markedly elevated at 12,061 U/L, and LDH was 893 U/L. Routine laboratory parameters, including kidney function tests, electrolytes, and complete blood count, were within normal limits. The chest X-ray showed no acute cardiopulmonary pathology.

Baseline 2D echocardiography demonstrated type I diastolic dysfunction with preserved left ventricular ejection fraction (55%) and no regional wall motion abnormalities.

**Figure 2:**
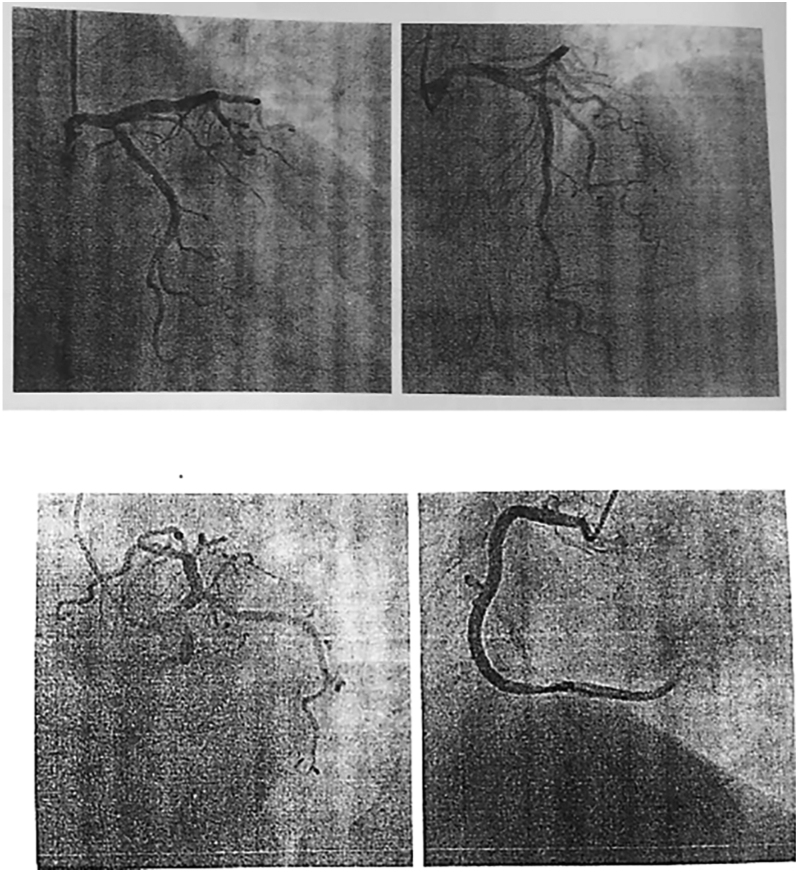
Coronary angiography revealing normal coronary arteries.

Coronary angiography was performed and did not reveal any significant coronary artery disease (Fig. 2). As per hospital protocol, thyroid function tests were also sent during the initial workup of chest pain. These revealed a markedly elevated TSH (>100 mIU/mL), low free thyroxine (T4) (0.08 ng/dL), and low free triiodothyronine (T3) (1.07 pg/mL). Anti-thyroid peroxidase (TPO) antibodies were elevated at 297.01 IU/mL, confirming autoimmune hypothyroidism.

During the hospital stay, serial cardiac biomarkers and repeat ECGs were obtained to monitor the clinical course. The repeated ECGs did not demonstrate dynamic ischemic changes characteristic of acute coronary syndromes, and serial cardiac enzyme levels, including CK-MB and troponin T, showed no extreme fluctuations.

## Treatment

The patient was diagnosed with severe hypothyroidism and was initiated on levothyroxine 100 μg daily, administered orally. Symptomatic management for chest pain included nitrates and routine monitoring for potential arrhythmias or heart failure exacerbation. He was also advised on lifestyle modifications, including maintaining smoking cessation and limiting alcohol intake.

## Outcome and follow-up

The patient showed clinical improvement during hospitalization and was discharged after stabilization. On outpatient follow-up after 2 months, he reported complete resolution of chest pain and dyspnea. Serial cardiac markers had normalized, and thyroid function tests demonstrated a TSH reduction to 5.5 mIU/ml, indicating effective hormonal replacement therapy. Repeat echocardiography was not performed due to financial constraints. The patient remained asymptomatic on continued levothyroxine therapy with no recurrence of symptoms.

## Discussion

This case illustrates the diagnostic complexity encountered when a patient presents with exertional chest pain, ischemic changes on electrocardiogram, and elevated cardiac biomarkers, yet is ultimately diagnosed with profound hypothyroidism in the absence of significant coronary artery disease.

Our patient exhibited classical features, suggestive of ACS, including anginal chest pain, diaphoresis, T-wave inversions on ECG, and elevated CK-MB and troponin T levels. Given this presentation, ACS was initially suspected, and urgent coronary angiography was performed. However, angiography revealed no significant coronary artery disease, and transthoracic echocardiography showed preserved left ventricular ejection fraction with no regional wall motion abnormalities. These findings, along with the absence of systemic illness and dynamic ECG changes, also made myocarditis unlikely, in accordance with the 2023 European Society of Cardiology (ESC) guidelines for ACS (1).

In such scenarios, it is important to consider non-coronary causes of cardiac biomarker elevation. Hypothyroidism is a recognized but frequently underappreciated cause of elevated CK-MB and troponin levels (2, 3, 4). Although hypothyroid myopathy often presents with symptoms, such as generalized weakness or muscle cramps, our patient reported no such complaints. His presentation was entirely cardiac in nature, which made the diagnosis more challenging and underscores the atypical manifestation of hypothyroidism in this case.

The underlying mechanisms of biomarker elevation in hypothyroidism are multifactorial. Thyroid hormones regulate mitochondrial biogenesis, myocardial energetics, and calcium homeostasis. Reduced levels of triiodothyronine impair oxidative phosphorylation and calcium cycling, leading to subclinical cardiomyocyte injury and leakage of intracellular enzymes, such as CK-MB and troponin, even in the absence of overt myocardial ischemia (5, 6, 7, 8). In addition, hypothyroidism may increase sarcolemmal membrane permeability, leading to muscle fiber breakdown and elevated serum creatine kinase, even in the absence of clinical myopathy (2). Furthermore, hypothyroidism-induced diastolic dysfunction, and fluid retention may elevate myocardial wall stress, potentially exacerbating subclinical enzyme leakage (9).

Other potential explanations for the observed biochemical pattern were considered. Macro-creatine kinase (macro-CK), a high-molecular-weight enzyme complex that can interfere with laboratory assays, was considered as a possible explanation (10). However, the normalization of enzyme levels with thyroid hormone therapy and the absence of fluctuating patterns made this unlikely. Euthyroid sick syndrome, a condition characterized by abnormal thyroid function tests during critical illness, was also deemed unlikely due to the absence of systemic illness, the markedly elevated TSH and the presence of thyroid autoantibodies confirming primary hypothyroidism. This case reinforces the importance of considering thyroid dysfunction in the differential diagnosis.

## Conclusion

This case highlights that profound hypothyroidism can present with symptoms such as ECG changes, and elevated cardiac biomarkers (including troponin), strongly mimicking acute coronary syndrome, even in the absence of coronary artery disease. It underscores the critical importance of including thyroid function tests in the diagnostic evaluation of patients with atypical presentations of myocardial injury. Timely diagnosis and initiation of levothyroxine replacement therapy, as demonstrated by this patient’s rapid clinical and biochemical recovery, can prevent misdiagnosis, avoid unnecessary invasive procedures, and lead to complete resolution of symptoms.

## Declaration of interest

The authors declare that there is no conflict of interest that could be perceived as prejudicing the impartiality of the work reported.

## Funding

This work did not receive any specific grant from any funding agency in the public, commercial, or not-for-profit sector.

## Patient consent

Written informed consent for publication of their clinical details and clinical images was obtained from the patient.

## Author contribution statement

PA and AA contributed equally to the conception and design of the case report. PA oversaw the patient’s clinical management and drafted the initial manuscript, while AA contributed to the analysis of the clinical data and critically revised the manuscript. Both authors reviewed and approved the final manuscript.
